# Secondary Radiation-Induced Bone Tumours Demonstrate a High Degree of Genomic Instability Predictive of a Poor Prognosis

**DOI:** 10.2174/138920212802510420

**Published:** 2012-09

**Authors:** Christine Rümenapp, Jan Smida, Iria Gonzalez-Vasconcellos, Daniel Baumhoer, Bernard Malfoy, Nabila-Sandra Hadj-Hamou, Bahar Sanli-Bonazzi, Michaela Nathrath, Michael J Atkinson, Michael Rosemann

**Affiliations:** 1 Institute of Radiation Biology, Helmholtz-Center Munich, German Research Center for Environmental Health, Neuherberg, Germany; 2 Institute of Pathology, Helmholtz-Center Munich, German Research Center for Environmental Health, Neuherberg, Germany; a Current adress: Technische Universität München, Zentralinstitut für Medizintechnik (IMETUM), Garching, Germany; 3 Department of Pediatrics and Wilhelm Sander Sarcoma Treatment Unit, Technische Universität München and Pediatric Oncology Center, Munich, Germany; 4 Clinical Cooperation Group Osteosarcoma, Helmholtz-Center Munich, German; Research Center for Environmental Health, Neuherberg, Germany; 5 Bone Tumour Reference Center at the Institute of Pathology, University Hospital Basel, Basel, Switzerland; 6 Institut Curie, Centre de Recherche, Paris, France; 7 CNRS, UMR3244, Paris France; 8 Université Paris VI, Paris France; 9 Department of Radiation Oncology, Klinikum rechts der Isar, Technische Universität München, Munich, Germany

**Keywords:** Genomic instability, Loss-of-heterozygosity, Osteosarcom, Predictive assay, Radiation-induced, SNP-array, Therapy-related, Therapy response.

## Abstract

Secondary bone tumours arising in the field of a preceding radiotherapy are a serious late effect, in particular considering the increasing survival times in patients treated for paediatric malignancies. In general, therapy associated tumours are known to show a more aggressive behaviour and a limited response to chemotherapy compared with their primary counterparts. It is not clear however whether this less favourable outcome is caused by inherent genetic factors of the tumour cells or by a general systemic condition of the patient. To elucidate this we analysed a series of bone sarcomas with a history of prior irradiation for the presence of genomic alterations and compared them with the alterations identified earlier in primary osteosarcomas. We analysed seven radiation induced bone sarcomas for genome-wide losses of heterozygosity (LOH) using Affymetrix 10K2 high-density single nucleotide polymorphism (SNP) arrays. Additionally, copy number changes were analysed at two distinct loci on 10q that were recently found to be of major prognostic significance in primary osteosarcomas. All the investigated tumours showed a LOH at 10q21.1 with 86% of cases (6/7) revealing a total genome-wide LOH score above 2400 and more than 24% of the genome being affected. Our results indicate similar genetic alterations in radiation induced sarcomas of bone and primary osteosarcomas with a poor prognosis. We speculate that the high degree of genomic instability found in these tumours causes the poor prognosis irrespective of the initiating event.

## INTRODUCTION

Exposure to ionising radiation is a risk factor for the occurrence of mesenchymal tumours of bone. A dramatic increase in the incidence of osteosarcomas (OS) and undifferentiated sarcomas (US) are observed in cases of occupational [1, 2] or medical exposure [3, 4] to high doses of osteotropic radio-isotopes. Whereas sub-lethal doses of radiation from an external source generally do not induce bone tumours, high-dose megavolt radiotherapy is known to be a risk factor for secondary bone tumour development [5-9]. These tumours are rare with an estimated risk of developing sarcomas in irradiated bone of 0.03-0.8% [10, 11]. As is the case for other types of therapy-associated secondary tumours, most studies reported a worse response to chemotherapy and a less favourable outcome compared to primary bone sarcomas having comparable morphology [10-14]. In a recent study it was shown that the prognosis of radiation-induced secondary sarcomas of bone (RSSB) can be improved when the current therapeutic protocols for primary bone tumours are applied [15]. The question remains however, whether the poor therapy response of RSSB to a standard therapy is due to their radiation-etiology, or if specific genetic alterations in the tumour cells are causing the poor response to therapy irrespective of the initiating event. 

We have recently shown that a combination of the degree of allelic imbalances and DNA copy number changes, together with specific changes at 10q21.1 and amplifications at 6p21, 8q24 and 12q14 yields a chromosomal alteration staging (CAS) system that discriminates between tumours with a good or poor chemotherapy response. The power of this system to predict therapy outcome was superior to histological assessment of tumour sensitivity at the end of the neoadjuvant chemotherapy [16]. To investigate if radiation induced OS carry similar genetic alterations as primary osteosarcomas, we now analysed a small set of secondary bone sarcomas that developed after radiotherapy. 

## MATERIALS AND METHODS

Archival samples from 7 bone sarcomas that developed within the field of a prior cancer radiotherapy regime have been investigated in this study. Of these, four were diagnosed as secondary high-grade osteosarcomas (OS, latency between 7 and 13 years after irradiation, patient age at the time of radiotherapy 4-14 months). The other three were diagnosed as undifferentiated sarcomas (US) due to the absence of unequivocal tumour osteoid formation (latency between 10 and 32 years, patient age at the time of radiotherapy 6 months to 54 years). The clinico-pathological characteristics of all investigated cases are listed in (Table **1**). Data concerning the individual therapy protocols, response of the RSSB to therapy and their clinical follow-up are not available.

Tumour DNA was extracted from 4 fresh-frozen pre-therapeutic biopsy samples (FFT) and 3 tumour xenografts (XG) and paired normal DNA from peripheral lymphocytes using a Quiagen DNAamp mini kit (Quiagen S.A. Courtaboeuf, France). Genome-wide losses of heterozygosity were analysed by hybridisation of the DNA onto Affymetrix 10K2 high-density single nucleotide polymorphism (SNP) arrays as described previously [16]. Loss of heterozygosity (LOH) was evaluated by directly comparing SNP genotypes in normal and tumour DNA and by statistically evaluating the length of uninterrupted homozygote intervals for the likelihood of such a pattern in the normal germline (yielding a LOH score). Copy number alterations on chromosome 10q were determined for two candidate gene loci (FGFBP3 and KIF11) using genomic qPCR (TaqMan, Applied Biosystems). C_T_ values of the loci of interest were converted into copy number alterations using the ΔΔ C_T _- method relative to paired lymphocyte DNA. For normalisation, two reference genes (GIT2 and ANP32d) on chromosome 12 were used, because their loci were very rarely affected by copy number changes. 

## RESULTS

### Extent of LOH in Radiation Induced Bone Sarcomas

All seven tumours exhibited a high degree of allelic losses. On average, 31.9% of the SNP markers of the tumours (range 12% - 55.8%) were part of long contiguous homozygote intervals with LOH-scores > 3 Fig. (**1A**, Table **1**). SNPs that mapped on these intervals and had a heterozygote genotype in the patients normal DNA exhibit a mono-allelic loss in the tumour, confirming that the calculated LOH-score is a reliable surrogate for a direct comparison of genotypes in normal and tumour DNA. In 6 of the 7 investigated tumours (or 86%), the total number of SNPs contributing to regions of LOH exceeded 2400 Fig. (**1B**, Table **1**). This is far beyond the cut-off of 1500 with which we predict good and poor responders in spontaneous OS cases [16].

### LOH Patterns and Copy Number Changes at 10q21.1 in Radiation Induced Bone Sarcomas

The only chromosomal locus that was affected by LOH in all the investigated cases was on chromosome 10q.21.1 Fig. (**2**). Quantitative real-time PCR for two genes on this locus (FGFBP3 and KIF11) confirmed copy-number losses in 4 out of the 7 cases, 2 cases had a copy number loss only relative to one reference locus, whereas only one tumour (no. 3) had no indication of copy number alterations at all (Table **1**). Allelic losses at 10q21.1 were already reported to be the single most powerful predictive marker associated with poor chemotherapy response and prognosis of spontaneous OS (LOH in 57 % of poor responders but in only 23% of good responders) [16].

### QC Information

The average genotype call rate was 94% in the lymphocyte (range 90.74% - 97.7%) and 85.15% in the tumour samples (range 79.3 % - 91.3%). The average percentage of SNP heterozygosity in patients normal tissue was 34.14% (range 33.3% - 34.98%) in agreement with the expect value of this marker set in the normal population.

## DISCUSSION

Secondary bone tumours are a rare late complication after successful tumour radiotherapy [10, 11]. However, since children increasingly survive treatment for paediatric malignancies the prevalence of post-radiation osteosarcomas is rising [17, 18]. Several studies have reported more frequent adverse outcome for patients with secondary bone sarcomas, compared to idiopathic primary cases [10-14]. We have recently proposed a chromosomal alteration staging system (CAS) that discriminates primary high-grade OS according to their clinical behaviour [16]. This allows improved prediction of the response to neoadjuvant treatment and more accurate overall prognosis prediction of individual patients at the time of initial diagnosis [16]. The recurrent alterations defining the CAS system included a genome-wide total LOH score > 1500, LOH on 10q and amplifications of 6p, 8q and 12q. 

In the present study we investigated seven radiotherapy associated secondary bone sarcomas for genome-wide LOH and copy number alterations to determine if these tumours harbour comparable genomic abnormalities than spontaneous cases. Our results show that all the investigated sarcomas exhibited a high degree of global allelic imbalances with on average 3481 out of 9769 SNPs being affected by LOH (N_LOH_). In the CAS protocol reported in our recent study of sporadic OS we found that a threshold of 1500 N_LOH_ yields the best discrimination between good and poor responders to chemotherapy [16]. The majority of cases presented here are therefore placed by CAS in the upper 50% of the reference group of poor responders and outside of the central 50% quartile of good responders. Radiation induced bone sarcomas develop global genomic instability to a similar extent as primary OS. The extent of the allelic imbalances, however, resembles that of primary OS with a poor response to chemotherapy. As reasoned elsewhere, a large degree of DNA gains and losses or allelic imballances is indicative of an ongoing genomic instability in cancer cells and probably contributes to the plasticity of tumour cells while escaping therapeutic intervention [19].

The single locus in the CAS system with the best discriminative power between good and poor responding primary OS was identified at 10q21.1 (LOH in 57% of poor responders but in only 23% of good responders) [16]. Interestingly, all of the secondary tumours included in the present study revealed LOH at this specific locus. This again suggests that secondary bone sarcomas do not follow a fundamentally different molecular pathway than their primary counterparts, but show genetic alterations to an extend that resemble those of poor responding primary cases. Radiotherapy induced OS were already reported elsewhere to harbour significant genomic instability, but this was not discussed as a molecular marker for therapy response or prognosis so far [20]. The few cases of RSSBs analysed here don’t exhibit an association between patient age and degree of genomic instability: the tumours with the highest extent of global LOH (case 6 and 7) were found in two patients from extremely different age groups (9.5 years and 75 years). A similar observation is made for two cases showing the lowest extent of LOH (case 1 and 2), which again arose in patients of very dissimilar age (12 years and 32.5 years). This contradicts the possibility that genomic instability is simply an age-related feature and therefore more prominent in secondary tumours in elder patients. In the tumours analysed here this phenomenon seems rather to be driven by the initiating event, in our case ionising radiation. 

Taken together our study suggests that common and recurrent genetic alterations exist in primary and secondary sarcomas of bone, indicating similarities in tumour development and/or pathways of malignancy. But the few cases of radiation-induced secondary sarcomas of bone analysed here demonstrate that they are over proportional frequently affected by a high degree of global allelic losses and by copy number alterations and LOH at 10q21.1. Considering that bone sarcomas with this etiology are very rare, larger studies on pre-therapeutic biopsy samples will always be extremely difficult to carry out. As a result of improved long-term survival after an initial radiotherapy, however the number of patients at risk to develop RSSBs later in life will rise. We therefore believe that for a better understanding of the molecular features of these unique tumour types and consequently for an optimised therapy one should also consider studies based on small patient numbers.

## Figures and Tables

**Fig. (1):**
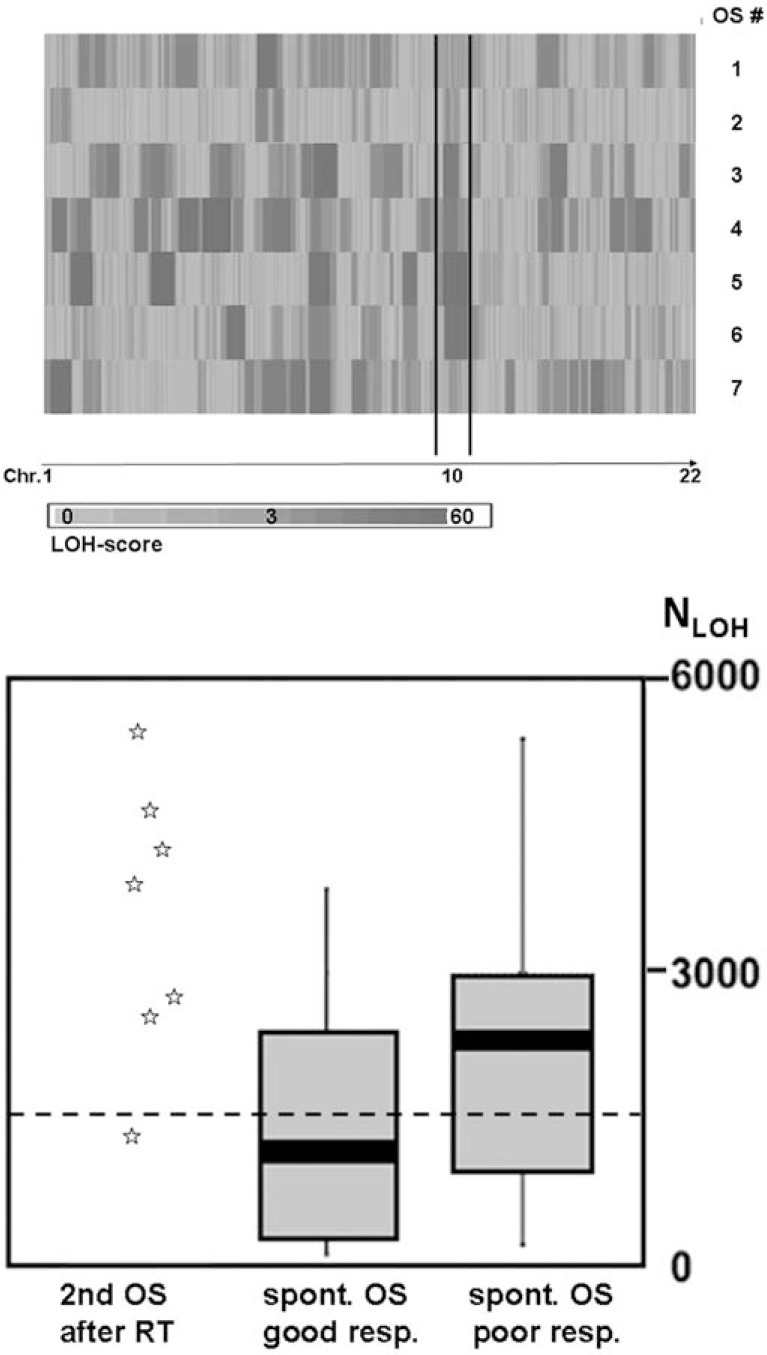
Extent of genome-wide LOH in 7 radiation-induced secondary sarcomas of bone. Pattern of chromosomal loci affected by LOH in the investigated sarcomas (**A**) and total number of SNPs with a LOH score > 3 in RSSBs as compared to primary osteosarcomas with good and poor response to chemotherapy (**B**). Dashed line at 1500 marks the best fitted discriminator between good and poor responding OS according to [16].

**Fig. (2):**
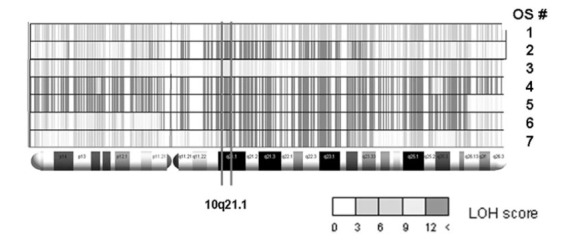
Pattern of LOH in the radiation-induced bone sarcomas along chromosome 10. Note that 10q21.1 is affected by LOH in all seven cases.

**Table 1.** Clinico-Pathological Characteristics and Results of LOH Analysis

Case	1^st^ Tumor	2nd Tumor	Age at Rx-Th	Latency (Years)	DNA Source	Number of SNPs affected by LOH*	Percentage of SNPs with LOH score > 3	Copy-Number Loss at 10q21.1
1	Retinoblastoma	OS	4 m	11	XG	1173	12.04	x
2	Retinoblastoma	US	6 m	32	XG	2412	24.7	x
3	Breast Cancer	US	50 a	10	FFT	2695	27.67	--
4	Retinoblastoma	OS	14 m	7	FFT	3797	39	(x)
5	Retinoblastoma	OS	13 m	13	FFT	4240	43.5	(x)
6	Laryngeal CA	US	54 a	21	XG	4618	47.4	x
7	Retinoblastoma	OS	8 m	9	FFT	5433	55.8	x

Legend:

* based on calculated LOH score

x DNA copy numbers in tumor reduced to < 0.7,

-- no detectable copy number change,

(x) copy number change only for one of the two reference loci.

## ABBREVIATIONS

LOH: = Loss-of-Heterozygosity
SNP: = Single-Nucleotide Polymorphism
RSSB: = Radiation-Induced Secondary Sarcoma of Bone
OS: = Osteosarcoma
US: = Undifferentiated Sarcoma
CAS: = Chromosomal Alteration Staging System
qPCR: = Quantitative Real-Time PCR
C_T_: = Threshold Cycle-Number (Used in qPCR)
QC: = Quality Control
